# Commentary: Prolactin Alters Blood Pressure by Modulating the Activity of Endothelial Nitric Oxide Synthase

**DOI:** 10.3389/fendo.2017.00105

**Published:** 2017-05-22

**Authors:** Jakob Triebel, Carmen Clapp, Gonzalo Martínez de la Escalera, Thomas Bertsch

**Affiliations:** ^1^Institute for Clinical Chemistry, Laboratory Medicine and Transfusion Medicine, Nuremberg General Hospital, Paracelsus Medical University, Nuremberg, Germany; ^2^Instituto de Neurobiología, Campus UNAM-Juriquilla, Universidad Nacional Autónoma de México (UNAM), Querétaro, México

**Keywords:** prolactin, vasoinhibins, 16K PRL, blood pressure, prolactin/vasoinhibin axis

Chang et al. report the modulation of blood pressure by prolactin and one of its cleaved products, a 16 kDa vasoinhibin isoform also referred as 16 kDa prolactin (1). They use transgenic mice producing prolactin in the liver and show that overexpression of the prolactin transgene leads to higher circulating levels of the vasoinhibin, which upregulates blood pressure by modulating the activity of endothelial nitric oxide synthase (eNOS). The high hepatic prolactin production leads to a surge in plasma prolactin levels. The circulating vasoinhibin might also originate in the liver, as the generation of the 16 kDa vasoinhibin isoform has been documented in rodent liver (2). Alternatively, the vasoinhibin could have been generated at a site other than the liver, as has been observed in various other organs and tissues such as the pituitary gland, heart, kidney, and vascular endothelium [reviewed in Ref. (3)]. Analysis of vasoinhibins in tissue homogenates and blood samples drawn from multiple veins may help answer this question.

The vasoinhibin isoform in the plasma of transgenic mice overproducing hepatic prolactin was characterized by Western blot analysis. The apparent molecular mass of 16 kDa was assigned presumably based on a molecular mass marker co-migrating in the SDS-PAGE. Prolactin cleaved by cathepsin D between tyrosine 147 and proline 148 would have a theoretical mass of 16.8 kDa (4). However, due to a lack of precision in the molecular mass determination, the band observed could also correspond to the 17.2 kDa cathepsin D-cleaved product (cleavage site: tryptophan150—serine151) (4) or the matrix metalloproteinase (MMP)-cleaved vasoinhibin isoform (cleavage site: serine155—leucine156) (5), or a mixture of both. This is relevant, as the composition of vasoinhibin isoforms varies among reproductive hypertensive diseases (3, 6). Nevertheless, the result shown in the current analysis is informative, as it indicates little or no generation of other vasoinhibin isoforms by cathepsin D or MMP (11, 12.5, 14.1, 15, and 17.7 kDa) in this animal model. The question of whether the 16 kDa vasoinhibin is a greater proportion of the total prolactin or reflects higher total prolactin could be answered by evaluating the optical density of prolactin and vasoinhibin values, provided the image is scaled to an intensity at which the prolactin signal is not oversaturated and the vasoinhibin signal is still visible.

The data suggesting that hyperprolactinemia results in higher circulating vasoinhibin levels which, in turn, induce plasminogen activator inhibitor-1 expression, lower eNOS phosphorylation/activation, and reduce nitric oxide production are novel, and they complement information regarding endocrine circuits in the prolactin/vasoinhibin axis and their relevance for cardiovascular function (3). Emphasis should be placed on evaluating circulating vasoinhibins when testing pregnant women for abnormally high prolactin levels. This is because of the wide range (35–600 ng/ml) of hyperprolactinemia values occurring in pregnancy (7) and the unclear correlation between circulating prolactin and pre-eclampsia (6).

## Author Contributions

JT wrote the manuscript. CC, GE, and TB revised the manuscript.

## Conflict of Interest Statement

The authors declare that the research was conducted in the absence of any commercial or financial relationships that could be construed as a potential conflict of interest.
